# Metagenomic Insights Into the Microbial Iron Cycle of Subseafloor Habitats

**DOI:** 10.3389/fmicb.2021.667944

**Published:** 2021-09-03

**Authors:** Arkadiy I. Garber, Ashley B. Cohen, Kenneth H. Nealson, Gustavo A. Ramírez, Roman A. Barco, Tristan C. Enzingmüller-Bleyl, Michelle M. Gehringer, Nancy Merino

**Affiliations:** ^1^School of Life Sciences, Arizona State University, Tempe, AZ, United States; ^2^School of Marine and Atmospheric Sciences, Stony Brook University, Stony Brook, NY, United States; ^3^Department of Earth Sciences, University of Southern California, Los Angeles, CA, United States; ^4^Department of Marine Biology, Leon H. Charney School of Marine Sciences, University of Haifa, Haifa, Israel; ^5^College of Veterinary Medicine, Western University of Health Sciences, Pomona, CA, United States; ^6^Department of Microbiology, Technical University of Kaiserslautern, Kaiserslautern, Germany; ^7^Biosciences & Biotechnology Division, Lawrence Livermore National Laboratory, Livermore, CA, United States

**Keywords:** metagenomics, marine sediment, marine aquifer, FeGenie, iron cycling, iron acquisition, iron oxidation, iron reduction

## Abstract

Microbial iron cycling influences the flux of major nutrients in the environment (e.g., through the adsorptive capacity of iron oxides) and includes biotically induced iron oxidation and reduction processes. The ecological extent of microbial iron cycling is not well understood, even with increased sequencing efforts, in part due to limitations in gene annotation pipelines and limitations in experimental studies linking phenotype to genotype. This is particularly true for the marine subseafloor, which remains undersampled, but represents the largest contiguous habitat on Earth. To address this limitation, we used FeGenie, a database and bioinformatics tool that identifies microbial iron cycling genes and enables the development of testable hypotheses on the biogeochemical cycling of iron. Herein, we survey the microbial iron cycle in diverse subseafloor habitats, including sediment-buried crustal aquifers, as well as surficial and deep sediments. We inferred the genetic potential for iron redox cycling in 32 of the 46 metagenomes included in our analysis, demonstrating the prevalence of these activities across underexplored subseafloor ecosystems. We show that while some processes (e.g., iron uptake and storage, siderophore transport potential, and iron gene regulation) are near-universal, others (e.g., iron reduction/oxidation, siderophore synthesis, and magnetosome formation) are dependent on local redox and nutrient status. Additionally, we detected niche-specific differences in strategies used for dissimilatory iron reduction, suggesting that geochemical constraints likely play an important role in dictating the dominant mechanisms for iron cycling. Overall, our survey advances the known distribution, magnitude, and potential ecological impact of microbe-mediated iron cycling and utilization in sub-benthic ecosystems.

## Introduction

Iron is the dominant redox-active element in the Earth’s crust and an important nutrient for almost all known life. In many environments, iron cycling is intimately linked to biogeochemical cycling of other elements, including carbon (e.g., CO_2_, CH_4_, and organic carbon), nitrogen (Laufer et al., 2016b; McAllister et al., 2020b), and heavy metals (Cooper et al., 2006). Thus, even though biologically available iron is comparatively rare/transient in many ecosystems, its speciation and flux considerably impacts the overall activities and productivity of diverse ecosystems. Research on the genetic basis for microbial iron cycling is in its infancy, and annotation pipelines annotate genes related to iron oxidation or reduction as “hypothetical” or simply “cytochrome c.” Accordingly, the extent of information that can be gained from metagenomes or metagenome-assembled genomes (MAGs), derived from shotgun sequencing, in relation to the potential for microbial iron redox cycling in the environment remains poorly constrained. This is particularly true of the marine subsurface, an extremely remote and difficult to access/sample environment, that is nevertheless significantly influenced by microbe-mineral interactions, particularly those related to iron oxidation and reduction (Edwards et al., 2003a; Roden, 2012).

While there have been studies, largely in ecosystems where iron-oxidizing and –reducing bacteria are conspicuously present (Riedinger et al., 2014; Laufer et al., 2016a,b; Beam et al., 2018; Bryce et al., 2018; Aromokeye et al., 2020; McAllister et al., 2020b), the extent of microbial iron cycling in other environments, including marine sediment and sediment-buried crustal aquifers, remains comparatively underexplored. Even though microbes capable of iron redox can form only a small proportion of the community in the latter habitats (e.g., rare biosphere), their influence on iron-cycling has potential to significantly impact the surrounding geochemistry. We previously developed FeGenie (Garber et al., 2020), a database and bioinformatics tool, to aid in annotating the iron redox genes and other genes involved in many aspects of the microbial iron cycle, including iron transport, storage, and regulation; we are also continuously updating the library of iron genes to include more genes and processes, such as siderophore transport and biosynthesis (Neilands, 1995), and fermentative iron reduction (Jones et al., 1984), which will be included in the next release. Herein, we used FeGenie, with an updated set of hidden Markov models (HMMs) for iron redox cycling and iron transport (Supplementary Table 1), to systematically profile the microbial iron cycle in recently published metagenomes representing six marine sediment sites and two sediment-buried crustal aquifer sites (Table 1). These metagenomes, published over the last decade, have recently illuminated the microbial lifestyle under the often harsh conditions imposed by subsurface geochemical regimes. Some of the original analyses of these metagenomes provided evidence for microbial iron oxidation and reduction occurring in the subseafloor, but these conclusions were inferred by using a limited iron redox gene database (Meyer et al., 2016; Tully and Heidelberg, 2016; Tully et al., 2018; Smith et al., 2019). Using FeGenie, we re-analyzed these metagenomes with a focus on iron cycling, using a standardized approach that includes all known genetic markers for dissimilatory iron reduction and oxidation. We note that these metagenomes were generated using a variety of wet-lab and *in silico* methods, limiting the cross-comparisons that can be carried out. Nonetheless, we highlight the potential for microbial iron cycling in the marine subsurface and demonstrate FeGenie’s capability to provide added valuable insights into iron cycling and acquisition/storage mechanisms in subseafloor habitats.

**TABLE 1:** Metadata for metagenome samples in which iron redox genes were detected with FeGenie.

Location	Longitude	Latitude	Depth below seafloor (bsf)	Depth below sealevel	NCBI BioProject ID	Metagenome references	Iron content	Iron references
Juan de Fuca U1362A (R/V Atlantis cruise ATL18-07)	47.761	–127.761	428–527 m (193–292 m below basement)	2,650 m	PRJNA269163	Jungbluth et al., 2017	0–1.1 μM aqueous Fe	Jungbluth et al., 2016
Juan de Fuca U1362B (R/V Atlantis cruise ATL18-07)	47.758	–127.762	264–352 m (29–117 m below basement)	2,650 m	PRJNA269163	Jungbluth et al., 2017	0–1.1 μM aqueous Fe	Jungbluth et al., 2016
Juan de Fuca 1301A (olivine biofilm)	47.754	–127.764	275–287 m	2,650 m	PRJNA264811	Smith et al., 2019	NA	NA
North Pond U1382A	22.750	–46.083	191.4–311.4 m (90–210 m below basement)	∼4,500 m	PRJNA391950	Tully et al., 2018	<1 μM Fe(II)	Bach, 2016; Meyer et al., 2016
North Pond U1383C	22.750	–46.083	123.6–285.6 m (70–232 m below basement)	∼4,500 m	PRJNA391950	Tully et al., 2018	<1 μM Fe(II)	Bach, 2016; Meyer et al., 2016
South Pacific Gyre (Expedition Knox-02RR)	–39.310	–139.801	0–5 cm	5,283 m	PRJNA297058	Tully and Heidelberg, 2016	5.6–7.3% Fe_2_O_3_	D’Hondt et al., 2011; Dunlea, 2016
Arctic mid-ocean ridge (Loki’s Castle)	73.763	8.464	3-11 m	∼3,250 m	PRJNA504765	Dharamshi et al., 2020	0–220 μM Fe(II) in pore water	Jørgensen et al., 2012, 2013
Costa Rica (IODP Expedition 334: U1378)	8.592	–84.077	2–93 m	∼1,000 m	PRJEB11766	Martino et al., 2019; Farag et al., 2020	Non-iron bearing clays	Expedition. 344 Scientists, 2011
Guaymas Basin	27.006	–111.409	0–60 cm	∼2,000 m	PRJNA362212	Dombrowski et al., 2018	NA	NA
Adriatic Sea (MET2 sample)	45.062	13.652	30 cm	NA	PRJEB13497	Gacesa et al., 2018	NA	NA
The solent	50.714	–1.464	0–8 cm	23.9–31.7 m	PRJEB6766	Smith et al., 2015	NA	NA
Eastern Gulf of Mexico (Site E26)	26.590	87.510	0–20 cm	2,800 m	PRJNA485648	Dong et al., 2019; Li et al., 2020	NA	NA
Western Gulf of Mexico (Chapopote, Oily sediment in the vicinity of the asphalt volcano)	21.964	93.226	0–10 cm	2,925 m	PRJEB32776	Laso-Pérez et al., 2019; Li et al., 2020	NA	NA
Santa Monica Basin (Eastern Pacific Ocean)	33.789	118.668	0–12 cm	860 m	PRJNA431796	Scheller et al., 2016; Yu et al., 2018; Li et al., 2020	NA	NA
Håkon Mosby Mud Volcano	72.004	14.730	0–10 cm	1,250 m	PRJNA248084	Ruff et al., 2019; Li et al., 2020	NA	NA

*NA, not available.*

## Materials and Methods

### Data Acquisition and Assembly

Metagenome assemblies representing North Pond fluids, which were made available by Tully et al. (2018), were downloaded from figshare (see original publication for figshare link). For the Guaymas Basin metagenome, in lieu of an assembly, we downloaded the 551 MAGs published by Dombrowski et al. (2018), and concatenated the contigs. Thus, due to the great amount of data available from these MAGs, no unbinned fraction from that dataset was evaluated. For all other metagenomic datasets, listed in Table 1, raw metagenome reads were obtained using the SRA Toolkit (release 2.10.0, SRA Toolkit Development Team). Reads were quality trimmed using Trimmomatic v0.36 (minimum length = 36 bp, sliding window = 4 bp, minimum quality score = 15, adaptors used = ILLUMINACLIP:TruSeq3-PE:2:30:10) (Bolger et al., 2014), and assembled using Spades v3.13.0 (default k-mers, Bankevich et al., 2012). Metagenome assemblies were then subjected to FeGenie analysis (Garber et al., 2020). For those metagenomes where metagenome-assembled genomes (MAGs) were publically available (Guaymas Basin, Loki’s Castle, Eastern Gulf of Mexico, Juan de Fuca ridge aquifer fluids, Juan de Fuca ridge olivine biofilms, and North Pond aquifer fluids), those were downloaded and also analyzed with FeGenie.

### FeGenie Analysis

We used FeGenie to identify iron genes in metagenome assemblies and MAGs. FeGenie was run with the –meta flag, directing the gene-calling software Prodigal, part of the FeGenie pipeline, to use its metagenomic procedure. Iron redox genes from the FeGenie output files were extracted using a custom python script (Supplementary File 1) and organized into pathways. To identify the closest sequenced relatives of identified iron genes, the protein sequences were extracted from FeGenie and queried (e-value cutoff: 1E-6) against the non-redundant protein database (release 240) from the National Center for Biotechnology Information (NCBI) using DIAMOND v2.0.4.142 (Buchfink et al., 2015). The closest phylogenetic relatives were then inferred from the top 50 DIAMOND matches and summarized in Supplementary File 2.

Siderophore biosynthesis clusters that were identified with FeGenie were confirmed using AntiSMASH v.5 (Blin et al., 2019). Contigs containing putative siderophore cluster genes were extracted using the grep command and subsequently subjected to AntiSMASH analysis (–cb-general, –cb-subclusters, –cb-knownclusters, –clusterhmmer, –asf, all other parameters were default). Results were then visually inspected for confirmation of potential for siderophore synthesis.

The R packages ggplot2 (Wickham et al., 2020), ggdendro (de Vries and Ripley, 2020), and Pvclust (Suzuki and Shimodaira, 2006), were used to generate plots presented in this article.

#### Site Descriptions

##### North Pond

The ∼8 Mya North Pond aquifer lies beneath a Mid-Atlantic ridge sediment basin and is composed of basaltic rocks, an important source of iron in this habitat, which are subject to chemical weathering by seawater due to advective fluid flow (Langseth et al., 1992; Meyer et al., 2016). Major solid weathering products include iron and manganese oxides, as well as carbonate minerals. Although classified as an oxic environment, the periodic presence of anaerobes and marker genes for low-O_2_ metabolisms suggests the occurrence of anaerobic microenvironments and redox oscillations (Tully et al., 2018). The frequency of redox oscillations is likely related to the seawater residence time in fractures, as well as the redox-buffering capacity of the rocks, in which anaerobic zones may occur due to fluid stagnation and consumption of oxygen through reaction with reduced minerals (MacQuarrie and Mayer, 2005; Trinchero et al., 2019). In contrast, intersecting fractures may result in fluid mixing of deep and shallow seawater, leading to microbial hot spots supported by fluctuating O_2_ concentrations that are ideal for iron-reducing and -oxidizing bacteria, something that has been previously observed in a terrestrial fractured rock aquifer (Bochet et al., 2020).

##### Juan de Fuca

The ∼3.5 Mya Juan de Fuca (JdF) ridge crustal aquifer is a basalt-hosted habitat located along a mid-ocean ridge flank with hydrothermal fluid circulation (Jungbluth et al., 2016). In comparison to North Pond, the geochemical signature of JdF fluids is more representative of extensive water-rock interactions due to longer fluid residence times and elevated fluid temperatures (Edwards et al., 2012). Seawater recharge occurs slowly due to the impermeability of the surrounding sediment and spreads laterally along fractures. Along the flowpath, fluid is heated and reduced, interacting with the basaltic rock, and is subsequently further altered by diffusive exchange with the overlying sediment pore water. The latter is most intense along the strong redox gradient at the sediment-rock boundary, where fluids are enriched in Fe, Mn, ammonium, Si, and Ca, and depleted in nitrate and oxygen (Wheat et al., 2013). Iron enrichment is particularly substantial and can be approximately 3000-fold higher in concentration relative to seawater and other JdF fluids in the flow path (Wheat et al., 2013).

##### Marine sediments

The metabolic potential for iron oxidation and reduction in marine sediments, hosting an estimated 0.18–3.6% of Earth’s total living biomass (Kallmeyer et al., 2012), remains understudied. Potential for oxidized iron to serve as an important electron sink and contribute significantly to the breakdown of organic carbon in sediment was recognized as early as 1963 (Kamura et al., 1963; Takai et al., 1963a,b). More recently, it was determined that *Zetaproteobacteria* are rare within marine sediments (estimated global abundance of 0.11%) but can contribute ∼8 × 10^15^ g of Fe in sedimentary iron oxides annually (Beam et al., 2018). Here, to survey the genetic capacity for iron cycling across diverse sedimentary regimes, we examine 27 marine sediment metagenomes (from 10 geographical sites, Table 1), including a low productivity oligotrophic site in the South Pacific Gyre, and high productivity sites like the Arctic Mid-Ocean Ridge (Dharamshi et al., 2020), Costa Rica Margin (Martino et al., 2019), and Guaymas Basin (Dombrowski et al., 2018).

## Results and Discussion

### The Impact of Shifting Redox Conditions on Iron Cycling in the North Pond Aquifer

Recent work, including metagenomic, metatranscriptomic, and colonization/poised-electrode experiments, provide evidence for iron oxidation within the North Pond aquifer. Metagenomic (Tully et al., 2018) and metatranscriptomic (Seyler et al., 2020) studies revealed the presence of genes associated with iron oxidation, specifically, *cyc2* [encoding an outer membrane porin-cytochrome fusion; (Appia-Ayme et al., 1998; Castelle et al., 2008; Barco et al., 2015; He et al., 2017, Keffer et al., 2021)] and *foxE* (encoding a periplasmic cytochrome; Croal et al., 2007; Pereira et al., 2017). Tully et al. (2018) also reconstructed metagenome-assembled genomes (MAGs) affiliated with the iron-oxidizing Zetaproteobacteria, which are known to adapt to fluctuating O_2_ concentrations and advective flow regimes (Chiu et al., 2017; Blackwell et al., 2020), further solidifying the presence and significant contribution that iron-oxidizing bacteria make to the aquifer community. Recent mineral colonization and current generation on poised electrodes also demonstrated the presence of iron oxidizing bacteria and provided evidence for their ability to utilize insoluble electron acceptors (Jones et al., 2020).

**FIGURE 1:**
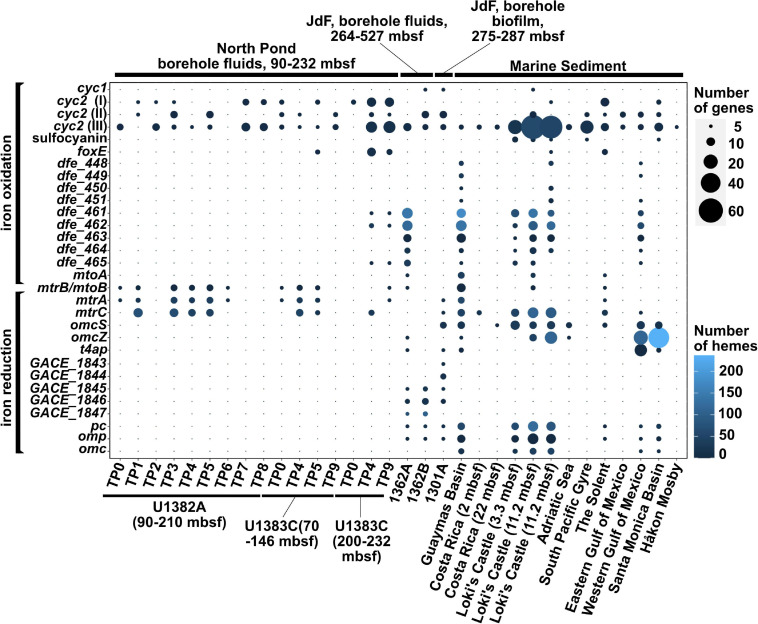
Distribution of iron redox genes among the 32 metagenomes in which potential for iron reduction or oxidation was detected. The gene *mtrB*/*mtoB* is deliberately placed in between the iron oxidation and iron reduction categories because it encodes a porin that can be part of both the iron reduction (*mtr*) or the iron oxidation (*mto*) pathways.

Our survey of 18 metagenome assemblies from the North Pond crustal aquifer (Tully et al., 2018) using the FeGenie library confirmed the presence of previously reported iron oxidases (Figure 1) (Tully et al., 2018; Seyler et al., 2020), which we phylogenetically linked to Zetaproteobacteria (*cyc2*) and Rhodospirillaceae (*foxE*) (Supplementary File 2). Linking *cyc2* to Zetaproteobacteria is important because this gene is highly diverse and is often encoded by taxa not known to be capable of iron oxidation; thus, only a handful of *cyc2* genes have been experimentally shown to be iron oxidases (Castelle et al., 2008; Jeans et al., 2008; Barco et al., 2015; McAllister et al., 2020a; Keffer et al., 2021). Further, we also documented the presence of genes associated with iron reduction [*mtrCAB* (Pitts et al., 2003; Hartshorne et al., 2007; Edwards et al., 2020) within seven of the timepoints, which span 2 years (Figure 1)]. The *mtrCAB* genes are most closely related to the dissimilatory iron reducer *Shewanella benthica* (Supplementary File 2), which was also enriched in mineral colonizations from the North Pond aquifer (Jones et al., 2020). We also identified nine MAGs (Tully et al., 2018), encoding copies of genes linked to respiratory iron oxidation or reduction (Supplementary Table 2). Notably, two MAGs that encode *cyc2* belong to the family Mariprofundaceae, known to encompass at least eight isolated strains of iron oxidizers (Emerson and Moyer, 2002; McBeth et al., 2011; Mumford et al., 2016; Barco et al., 2017; Chiu et al., 2017; McAllister et al., 2019). Another MAG, which belongs to the genus *Shewanella*, encodes two copies of the *mtrCAB* operon for iron reduction. Other putative iron oxidizers and reducers are shown in Supplementary Table 2. 16S rRNA gene amplicon sequencing presented by Jones et al. (2020), showed that *Geobacter*-related spp. were also enriched on minerals; however, we did not detect any *Geobacter*-related gene markers for iron reduction (*omcS*, *omcZ*, and type IV aromatic/electroactive pili [*t4ap*]), suggesting that this lineage may be part of the rare biosphere and undetectable with metagenomic approaches, at least in the aquifer fluids. Additionally, two metagenomes, derived from bottom water sampled near the North Pond aquifer, showed potential for iron oxidation via *cyc2*, *foxE*, and sulfocyanin, a blue-copper protein used as a genetic marker for iron oxidation in Archaea (Castelle et al., 2015). However, these sequences were not related to known iron-oxidizing bacteria (e.g., Mariprofundaceae), and no iron reduction genes were detected.

**FIGURE 2:**
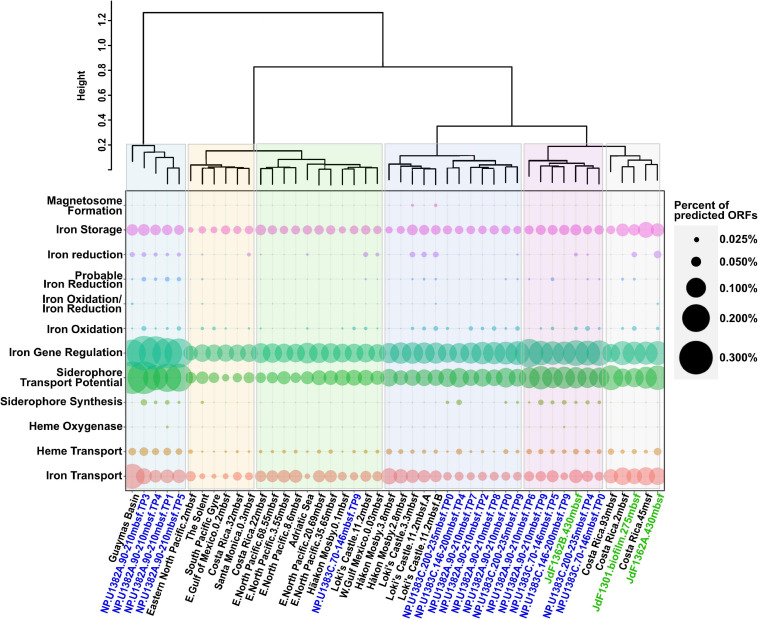
Dotplot summary of iron genes in marine subcrustal aquifers and sediment identified by FeGenie, showing the variation among iron genes from different time points from North Pond. North Pond metagenomes are colored blue, while metagenomes from the Juan de Fuca ridge are colored green. Metagenomes derived from sediment samples are colored black. While certain pathways appear to be universal (iron acquisition and storage), other processes appear to vary in abundance (iron redox cycling and siderophore synthesis), while others still (magnetosome formation and heme oxygenases) appear to be quite rare. “Percent of predicted ORFs” was calculated by dividing the total number of genes identified from each iron gene category by the total number of predicted ORFs from each respective metagenome.

The co-occurrence of iron oxidizers and reducers in the North Pond aquifer supports potential for coupling of iron redox processes. Mutualistic interactions between iron oxidizers and reducers have been reported in various habitats (Weber et al., 2006; Blöthe and Roden, 2009; Emerson, 2009; Roden et al., 2012; Elliott et al., 2014; Byrne et al., 2015). While the coupling of iron reduction with oxidation may be less apparent in an iron-rich habitat like the Loihi Seamount (Emerson and Moyer, 2002), iron reducers and iron oxidizers may be more dependent on each other’s metabolic by-products in the North Pond aquifer, where dissolved iron and carbon concentrations are much lower. As noted above, the presence of anaerobes within the North Pond aquifer hints at the possibility of sub-oxic microenvironments and varying redox conditions (Tully et al., 2018). This variability may be reflected in the observed fluctuation of iron oxidases and reductases over the 2-year sampling period (Figure 1). Consistent with this, hierarchical clustering of the metagenomes based on the overall iron gene composition (including iron acquisition and storage) demonstrated the dissimilarity between different North Pond timepoints (Figure 2), consistent with the observed temporal separation between iron reducers and oxidizers. Iron-oxidizers and reducers can take advantage of redox oscillations (Coby et al., 2011; Jewell et al., 2016), and in the North Pond aquifer, dominance of either iron reducers and oxidizers may be cyclical.

### Different Iron Redox Strategies Between Planktonic and Biofilm Communities of the Juan de Fuca Ridge

Evidence for iron oxidation at the JdF first came in the form of a chemolithoautotrophic iron-oxidizing isolate (Edwards et al., 2003b), and then with the identification of biogenic iron oxides resembling those formed by iron-oxidizing bacteria, like *Mariprofundus* and *Leptothrix* (Kennedy et al., 2003; Toner et al., 2008). However, this evidence is from seafloor samples collected in the vicinity of the JdF aquifer. For example, the biogenic iron oxides were either (i) found in iron oxide mounds (Kennedy et al., 2003) or (ii) enriched from mineral incubations deployed at the seafloor (Toner et al., 2008). No sequence-based analysis has been performed on these iron oxide-rich samples, and the exact identity and mechanisms for iron-cycling in microbial communities associated with those iron oxides remains unknown. Three metagenomes representing the planktonic (2 samples) and crust-associated (1 sample) microbial communities from the sediment buried crustal aquifer at the JdF have since been published, allowing molecular interrogation of genetic potential for iron-related metabolisms. Two crustal aquifer planktonic metagenomes (Jungbluth et al., 2017) were collected at subseafloor observatories retrofitted with Circulation Obviation Retrofit Kits [CORKs; Edwards et al. (2012)]; one biofilm metagenome is derived from an olivine chip biofilm incubated *in situ* for 4 years within a Flow-through Osmotic Colonization System [FLOCS; Orcutt et al. (2010)] (Smith et al., 2019).

FeGenie analysis revealed the genetic potential for iron oxidation, via *cyc2*, in all three JdF metagenomes, while *mtoAB*, a larger multi-protein porin-cytochrome complex (Jiao and Newman, 2007; Liu J. et al., 2012), was detected in only one of the crustal fluid metagenomes (Figure 1). The JdF crustal biofilm was previously reported to not support iron oxidation through the analysis of MAGs alone (Smith et al., 2019). Analysis of the whole metagenome assembly, including the unbinned fraction (contigs that were not included in the MAGs), identified at least eight *cyc2* gene copies. Seven of these *cyc2* copies were in the unbinned fraction of the assembly, and one was detected in JdFRolivine-5, a MAG (within the class Clostridia) constructed and published by Smith et al. (2019). Despite its significant homology, the *cyc2* copy in JdFRolivine-5 has unclear function, as it is considerably shorter than most other *cyc2* genes.

Smith et al. (2019) also reported that the MAG JdFRolivine-10 is related to the known iron reducer *Geoglobus* (Slobodkina et al., 2009; Mardanov et al., 2015) and encodes some of the cytochromes implicated in iron reduction in *Geoglobus acetivorans* (Mardanov et al., 2015). Three multiheme cytochromes encoded by JdFRolivine-10 match those in FeGenie’s HMM library (Supplementary Table 2), which were previously linked to extracellular electron transfer: DFE_0449 (14-heme iron oxidase; Deng et al., 2018), GACE_1846 (4-heme iron reductase), and GACE_1847 (22-heme iron reductase) (Mardanov et al., 2015). The outer membrane cytochrome with locus tag GACE_1847 was predicted to encode an outer membrane anchor domain, as well as two hematite-binding sites (Mardanov et al., 2015). These cytochromes are all located on different contigs in JdFRolivine-10’s genome and were identified using the new FeGenie flag –all_results, allowing us to bypass FeGenie’s built-in operon-evaluating algorithm. FeGenie also confirmed the presence of a hematite-binding motif (a new feature in FeGenie’s pipeline, see Supplementary Methods for details). The presence of heme-binding and transmembrane domains encoded on the GACE_1847 homolog provides a clue to one of the possible mechanisms for iron reduction within the JdF aquifer biofilm. Other genes related to iron reduction were also detected in the unbinned fraction of the metagenome from JdF olivine biofilms: *omcS* (Mehta et al., 2005; Qian, 2011; Wang et al., 2019), *mtrC* (Hartshorne et al., 2007), *mtrA* (Pitts et al., 2003), and *t4ap* (Bray et al., 2020), further supporting the potential for iron reduction within crustal biofilms. However, since olivine contains only reduced iron (Fe^2+^), it likely has been oxidized first by iron-oxidizing bacteria, supporting a potential cryptic iron cycle on the surface of the olivine mineral (Supplementary Figure 1); and since JdF is an anoxic system, this implicates anaerobic iron oxidation, possibly nitrate-dependent.

In the crustal fluid metagenomes, we detected potential for iron reduction via *t4ap* (Bray et al., 2020), *omcZ* (Inoue et al., 2010), and the flavin-based extracellular electron transfer mechanism recently reported in *Listeria monocytogenes* (Light et al., 2018). Potential for iron oxidation was detected via the *dfeEFGHI* operon (Deng et al., 2018). Unlike the olivine biofilms, *omcS* was not detected in the fluid metagenomes. This gene is associated with *Geobacter* species (Qian, 2011; Wang et al., 2019), which prefer anaerobic environments and less likely to dominate fluids subject to redox gradients (Lin et al., 2004; Lovley et al., 2011; Engel et al., 2020). Indeed, four *omcS* copies detected in the biofilm were phylogenetically linked to *Geoalkalibacter subterraneus* and *Malonomonas rubra* (Supplementary File 2 and Supplementary Table 4), both obligate anaerobes that taxonomically fall within the *Desulfuromonadales* order, which also contains *Geobacter* spp. Because the planktonic community in the anoxic crustal fluids is sourced from oxic bottom waters, we hypothesize that these iron reducers, rare in seawater, remain rare in the chemically evolved anoxic fluids. Eventually, these lineages may transition from planktonic to biofilm lifestyles where the activities of previous microbial communities (e.g., iron-oxidizing and sulfur-reducing bacteria) provide them with oxidized iron substrates (Toner et al., 2008; Ramírez et al., 2016). For example, as mentioned above, we did not detect any *Geobacter*-related gene markers from the North Pond aquifer metagenomes; however, recent mineral incubations enriched an electroactive consortia that includes *Geobacter* (Jones et al., 2020), supporting the latent presence of this lineage in redox-fluctuating fluids.

### A Survey of Iron Redox Potential in Globally Dispered Marine Sediment

Potential for benthic iron reduction is prevalent among the locations under relatively high water-column productivity (e.g., Loki’s Castle, Guaymas Basin, and Western Gulf of Mexico), but is absent in the oligotrophic South Pacific Gyre (SPG) (Figure 1). The lack of iron reduction in SPG is consistent with previous analysis of these sediments (Tully and Heidelberg, 2016). High water column productivity results in benthic conditions ideal for iron reduction (low oxygen and high organic carbon deposition rates). The South Pacific Gyre sediments are far from this ideal, as they are low in carbon and oxic throughout the entire sediment column (D’Hondt et al., 2009). Two of the four cold-seep sediment metagenomes (Håkon Mosby Mud Volcano and Eastern Gulf of Mexico) also lacked any detectable genes for iron reduction. The surveyed metagenome sample from the Håkon Mosby Mud Volcano represents the top cm of young, freshly-erupted mud with ongoing aerobic activities, and, thus, is also not primed for iron reduction (Ruff et al., 2019). It is unclear why we did not detect genes for iron reduction in the Eastern Gulf of Mexico sediment (Dong et al., 2019); this site represent a petroleum seep where benthic communities may be dominated by hydrocarbon-degrading microbes that are not capable of iron reduction. It is important to note that technical (e.g., number of contigs, contig lengths, etc.) and environmental (community richness) differences can significantly influence inferences from broad metagenomic surveys; often, several factors (e.g., quality and concentration of DNA, strain-level diversity, prevalence of repetitive DNA sequences) can play a major role in determining the quality of the metagenome assemblies, as evidenced from the substantial variation in the distribution of contig lengths obtained from different metagenome assemblies (Supplementary File 3). This can affect the capacity of annotation pipelines, like FeGenie or AntiSMASH, to detect certain pathways.

Higher productivity sites demonstrated a diverse array of genes for iron reduction, including *omcS*, *omcZ*, and *mtrCAB*, etc. *Geobacter*-related iron reductases [e.g., *omcS*, *omcZ*, and other porin-cytochrome complexes (Shi et al., 2014)] were more common in the marine sediment sites compared with the surveyed aquifer metagenomes, with only the Guaymas Basin encoding the *mtrCAB* operon. Numerous copies of the aromatically dense type IV pili (Bray et al., 2020) gene were detected. These are particularly prevalent in marine sediment from the Western Gulf of Mexico (Laso-Pérez et al., 2019).

Potential for iron oxidation was detected in nearly all surveyed marine sediment sites (Figure 1). Specifically, *cyc2* [more specifically, *cyc2*-cluster 3 (McAllister et al., 2020a)] appears to be the most common gene putatively linked to iron oxidation. In the North Pond aquifer, many *cyc2* homologs were found to be closely related to those encoded by the iron-oxidizing *Mariprofundus*. However, in marine sediments, *cyc2* homologs appear to have a different phylogenetic distribution, with associated taxa that have not previously been linked to iron oxidation (e.g., Acidobacteria, Desulfobacterales, *Ralstonia solanacearum*, etc.). In these taxa with no experimental evidence for iron oxidation, the function of *cyc2* is less clear. While *cyc2* appears to be most widely distributed porin-cytochrome putative iron oxidase, *mtoAB* is also present in three sediment sites: Guaymas Basin, Loki’s Castle, and The Solent. Other genetic markers for iron oxidation were also detected: sulfocyanin was detected in three of the marine sediment sites (Loki’s Castle, South Pacific Gyre, and Santa Monica Basin); the periplasmic cytochrome-encoding *foxE* was found in two sites (Loki’s Castle and The Solent). The Solent, which represents the only sediment metagenome from the photic zone, was found to have a variety of different markers for iron oxidation and reduction. Thus, unlike other sites that are well below the photic zone (Table 1), where iron oxidation has potential to fuel chemolithoautotrophy, in The Solent, iron oxidation may fuel photoferrotrophy. While the oxic South Pacific Gyre sediment may support aerobic iron oxidation, the other surficial sediment sites, with higher productivity and lower oxygen content, may support continuous iron oxidation at the surface or during periods of oxygenation [e.g., via bioturbation (Beam et al., 2018)].

### Magnetosome Formation at Loki’s Castle, but Absent From Other Sediment and Aquifer Metagenomes

Magnetosomes are organelles of magnetotactic bacteria that contain biomineralized magnetite (Frankel et al., 1979; Uebe and Schüler, 2016). This allows bacteria to sense the Earth’s magnetic field, facilitating orientation along geochemical gradients. We detected nine separate loci encoding genes for magnetosome formation at Loki’s Castle sediment (Figure 2). The genetic loci for magnetosome formation identified at Loki’s Castle presented a mixed phylogenetic distribution, with amino acid identities near 50%, likely indicating a lack of closely related sequenced organisms. However, some of the most closely related sequences in NCBI’s non-redundant protein database appear to be from the Magnetococcales order, which includes *Magnetococcus marinus*, a marine magnetotactic bacterium isolated from the oxic-anoxic sediment-boundary off the coast of Rhode Island (Bazylinski et al., 2013). Magnetosomes are conceivably advantageous in environments with geochemical gradients, and are considered to be ubiquitous in aquatic habitats (Lefèvre and Bazylinski, 2013). Thus, the apparent lack of magnetosome formation operons in most marine sediment and all aquifer metagenomes is somewhat surprising. It is possible that magenetosome formation is heavily dependent on the availability and speciation of iron, which varies as a function of the spatial distribution of the redox cascade. Alternatively, the dearth of magnetosome formation operons (*mam*) can be a result of FeGenie’s strict rules for its detection (requiring the presence of at least 5 of the 10 diagnostic genes); while the assembly qualities for most of the metagenomes used in our survey are high (Supplementary File 3), detection of magnetosome formation operons requires contigs greater than 5,000 bases in length, and these make up a relatively small proportion (∼0.1–20%) of contigs in each assembly. In support of the latter hypothesis, we identified a MAG with genetic potential for iron oxidation (via *foxE*) – this MAG, with a ∼75% complete genome, is closely related to the *mam*-encoding *Magnetovibrio blakemorei*, but only has 2/10 of the *mam* genes, which are found on two different contigs; this is below FeGenie’s detection threshold.

### Iron Acquisition and Storage Is Common, but Siderophore Synthesis Is Limited to Subsurface Aquifers and One Sediment Site

In addition to genes for iron redox cycling and magnetosome formation, we also report the distribution of genes for iron acquisition and storage, which are not directly linked to respiration, but are essential functions for the majority of living organisms (Figure 2). As expected, metagenomes examined in this study have the genes necessary for ferrous and ferric iron transport [*efeUOB, fbpAB(C), feoAB(C)*, *futABC*], iron storage within ferritin (PF00210), as well as iron gene regulation (*dtxR, fecR, feoC*, and *fur*) (Supplementary Table 3). Potential for transport of iron-chelating molecules like siderophores (Butler, 2005) and heme via the TonB-ExbB-ExbD system was also detected (Krewulak and Vogel, 2011). We note that TonB-dependent transport is not specific to iron-chelating compounds (Noinaj et al., 2010). Even though heme can also be transported by this pathway (Noinaj et al., 2010; Krewulak and Vogel, 2011), the marine sediment metagenomes were largely devoid of heme oxygenase and storage homologs, except for one copy of *pigA* (heme oxygenase; Friedman et al., 2003, 2004) and a few copies of *hutZ* (heme storage; Liu X. et al., 2012) in the North Pond metagenomes.

The North Pond metagenomes harbored several siderophore synthesis gene clusters that share similarity to the known clusters encoding desferrioxamine E, crochelin A, amonabactin P, vicibactin, and vibrioferrin (Supplementary Table 5). The percent of genes in these identified clusters with significant BLAST hits to genes within known siderophore clusters ranged from 33–80%, as determined by AntiSMASH (Blin et al., 2019). Putative siderophore synthesis was also confirmed in several North Pond MAGs (Supplementary Table 2). MAGs taxonomically identified as *Pseudomonas tetraodonis*, *Halomonas alkaliantarctica*, and *Paracoccus* sp. may produce siderophores related to desferrioxamine E (75% gene similarity), crochelin A (46%), and amonabactin P (42%), respectively. Other North Pond MAGs encode siderophore gene clusters similar to aerobactin (33%; *Moritella* sp000170855), bisucaberin B (100%; Flavobacteriaceae sp.), and unknown siderophores. The presence of siderophore synthesis clusters in the North Pond metagenomes and MAGs with low similarity to known siderophore biosynthesis genes suggests potential for synthesizing structurally related or novel siderophores. Siderophore clusters are genetically diverse and modular systems that are impacted by several evolutionary processes, including gene loss and gene acquisition (e.g., through horizontal gene transfer) (Cruz-Morales et al., 2017; Thode et al., 2018). This leads to variation in siderophore structures (e.g., Seyedsayamdost et al., 2012), which is likely required to prevent uptake by non-siderophore-producing microorganisms, in what is known as siderophore piracy (Hibbing et al., 2010; Butaitë et al., 2017).

We also detected a siderophore synthesis locus in one of the JdF aquifer fluid metagenomes (Supplementary Table 5). Using AntiSMASH, this cluster was confirmed to be related to the acinetoferrin biosynthesis cluster.

Out of the 24 surveyed metagenomes from marine sediment, only one, The Solent, appears to encode a siderophore biosynthesis cluster, confirmed using AntiSMASH, to be most closely related to xanthoferrin. The overall lack of siderophore production in marine sediments may be due to the greater bioavailability of iron in those habitatis, or the potential presence of siderophores derived from the water column. The metagenomes may also harbor siderophore synthesis loci that share no or undetectable similarity to known siderophore synthesis clusters/models. Siderophores are necessary to obtain insoluble Fe(III) from the environment, and ferrisiderophore complexes become an important source of iron when soluble Fe(II) is limited. At Loki’s Castle and Guaymas Basin sediments, pore water Fe(II) concentrations can reach ∼200 μM (Table 1), which may obviate the need to synthesize siderophores. In the South Pacific Gyre sediment, with extremely low productivity and metabolic activities, the synthesis of siderophores may be too energetically costly. In contrast, siderophore synthesis in North Pond and JdF fluids concur with the minimal amounts of bioavailable iron present (Table 1), although JdF fluids have been shown to have highly variable iron concentrations between boreholes (Wheat et al., 2013). Within North Pond, oxic conditions (∼213–216 μM O_2_) result in the biotic and abiotic removal of soluble iron, leading to extremely low iron concentrations. This is consistent with the relatively high numbers of different siderophore synthesis loci identified there. The apparent lack of siderophore production in iron-limited sediment, like Costa Rica sediments, mainly composed of non-iron bearing clays (Vannucchi et al., 2013), and South Pacific Gyre sediments, which are oxygenated and contain only 5.6–7.3% Fe_2_O_3_ (D’Hondt et al., 2011; Dunlea, 2016) suggests dependence on exogenous siderophores, or other iron-chelating molecules (D’Onofrio et al., 2010). For example, in higher-productivity areas, sedimentation of biomass may deliver sufficient amounts of iron and iron-bearing molecules to benthic communities (Boyd and Ellwood, 2010; Gledhill and Buck, 2012; Boiteau et al., 2016). Additionally, release of reduced iron from sediment, which can also be catalyzed by iron-reducing bacteria, can potentially contribute to the iron budget of resident microbes. Alternatively, as mentioned above with regard to the apparent lack of magnetosome formation operons, the lack of siderophore biosynthesis operons may also be due to the fact that, similar to the magnetosome formation operon, siderophore biosynthesis operons are often >10,000 bases in length and involve multiple genes; thus, reliable detection of siderophore biosynthesis requires contigs that are relatively rare in metagenome assemblies.

## Concluding Remarks

**FIGURE 3:**
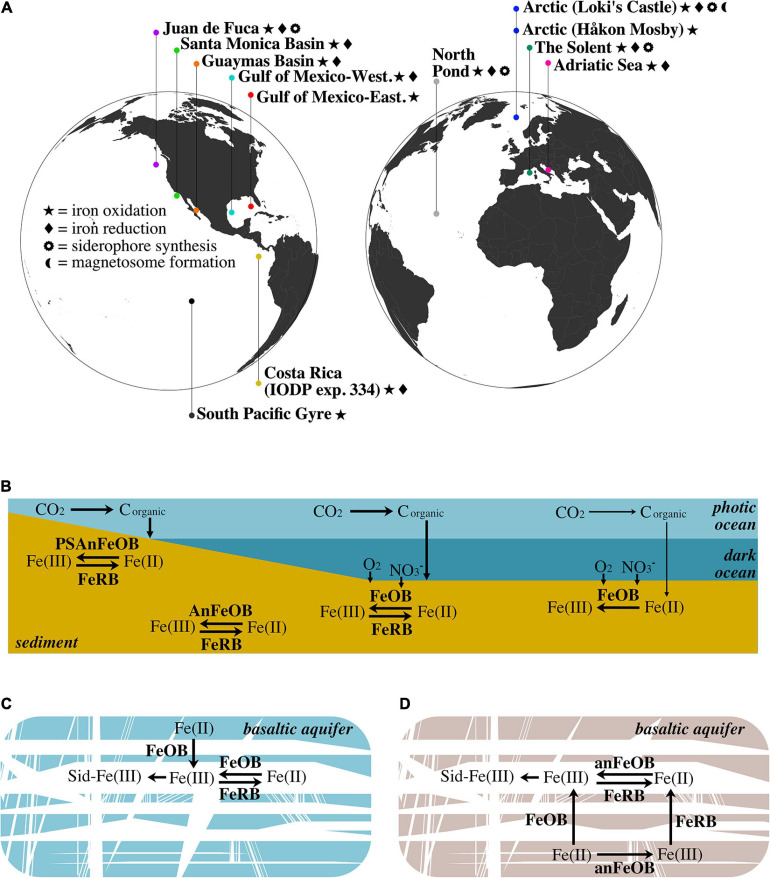
**(A)** Geographic distribution of marine aquifer and sediment sites from which metagenome samples are derived. **(B)** Schematic of the iron redox cycle in various types of marine sediment. In shallow sediment within the photic zone (e.g., The Solent), photoferrotrophy can drive iron oxidation. Below the photic zone, aerobic iron oxidation in surficial sediment (e.g., Guaymas Basin, Santa Monica Basin, and Western Gulf of Mexico) is contrasted with anaerobic iron oxidation (e.g., nitrate-dependent) in deep anoxic sediment (e.g., Costa Rica and Loki’s Castle). In these aforementioned sediments, iron reduction is present and represents the other half of the iron cycle. Conversely, in oxic sediments, such as those underlying areas of low productivity (e.g., South Pacific Gyre), aerobic iron oxidation is the dominant process, with no detectable iron reduction. The lower productivity is denoted by thinner arrows that signify carbon fixation and sedimentation. **(C)** Iron cycling within the cold and oxic North Pond aquifer: iron is released from the young, largely ferrous (Bach and Edwards, 2003), basaltic crust via iron oxidation, where it is free to cycle between the ferric and ferrous forms, and subject to the chelating activity of siderophores. **(D)** Iron cycling within the warm, anoxic JdF aquifer: since the waters circulating in this aquifer are anoxic, iron oxidation is likely dependent on anaerobic metabolisms. Similar to North Pond, JdF basaltic crust is young and the iron content is largely ferrous (Bach and Edwards, 2003); thus, iron is first released via oxidation. Detection of iron reduction on the surface of an olivine chip suggests that iron oxides may be deposited on the olivine mineral after oxidation by iron-oxidizing microbes. FeOB, aerobic iron-oxidizing bacteria; AnFeOB, anaerobic iron-oxidizing bacteria; PSAnFeOB, photosynthetic anaerobic iron-oxidizing bacteria; FeRB, iron-reducing bacteria; Sid, siderophores.

Our reanalysis of globally distributed marine subsurface metagenomes using FeGenie’s comprehensive iron gene library illuminates the diversity of microbial iron redox mechanisms that can occur across a range of geochemical regimes (Figure 3A). Further, we demonstrate FeGenie’s utility in providing a standardized pipeline for the comparison of iron genes among many large genomic datasets. We note that the data used in our survey were generated from multiple studies; inherent differences in sampling collection and processing, sequencing, and *in silico* methods, thus, make it difficult to generate overarching conclusions. Despite these potential limitations, our results support a hypothesis that geochemical constraints may influence the distribution of iron redox genes, potentially playing an important role in determining the dominant strategy for iron cycling (Figures 3B–D). At high-productivity sites (e.g., Guaymas Basin, Santa Monica Basin, Western Gulf of Mexico, Costa Rica), iron cycling is likely based on the penetration of oxygen and/or nitrate into surficial sediment, or deeper due to bioturbation, enabling microbial iron oxidation processes until these electron acceptors are diminished and iron reduction dominates. In oxic sediment, such as that underlying areas of low productivity (e.g., South Pacific Gyre), iron cycling is likely dominated by iron oxidation – with no or limited biological iron reduction. In cold and oxic subseafloor aquifers like North Pond, the flux of oxygen and organic carbon are possibly key factors influencing the abundances of iron-oxidizers and iron-reducers (Figure 3C), while in the warm and anoxic waters circulating within the JdF aquifer, iron cycling is dependent on anaerobic metabolisms (e.g., iron reduction and nitrate-dependent iron oxidation) (Figure 3D).

In nearly every site where iron redox cycling was detected, porin-cytochrome combinations were present and may constitute an important vector for dissimilatory electron transfer to and from soluble and insoluble iron (although porin-cytochrome genes are also the most common genetic markers currently available). For example, the porin-cytochrome fusion encoded by *cyc2* appears nearly ubiquitous in our surveyed data; *cyc2*, whose phylogenetic distribution implies rampant lateral transfer among prokaryotes (McAllister et al., 2020a), may thus represent a widespread mechanism for respiratory iron oxidation. Alternatively, it is possible that Cyc2 acts as an iron detoxification mechanism (Bradley et al., 2020). In addition to porin-cytochromes, other mechanisms for iron redox were detected in diverse habitats. For example, the *Geobacter*-type *omcS*, *omcZ*, and electroactive pili genes demonstrate another strategy that may be largely relegated to sediment and biofilm niches. Although, detection of *omcZ* and *t4ap* genes in one of the JdF aquifer plankton metagenomes supports the possibility that this strategy may be utilized in planktonic niches, at least those where anoxic conditions prevail.

Geochemical constraints also seem to impact other aspects of the microbial iron cycle. Unlike the near-ubiquitous occurrence of genes associated with iron transport, iron storage, iron gene regulation, heme transport, and siderophore transport amongst the surveyed metagenomes, siderophore biosynthesis gene clusters were restricted to subseafloor aquifers, with only one sediment site encoding a siderophore biosynthesis cluster. Similarly, magnetosome formation genes were detected in only one of the sediment metagenomes (and none in the subsurface aquifers). It is possible that these biosynthesis operons are in short supply due to geochemical factors influencing the supply of iron or energy needed to synthesize them. However, we also cannot rule out the possibility that siderophore biosynthesis and magnetosome formation are more prevalent but undetected, either due to high level of sequence divergence from pre-existing HMMs used by FeGenie, or due to low abundances in the sequenced samples. For example, FeGenie retains the ability to detect potentially novel types of siderophore biosynthesis clusters, but detection of magnetosome formation depends on gene markers from a single known operon. In summary, our survey provides a comprehensive overview using the currently available genetic markers, generating testable hypotheses and providing insights into the distribution of iron genes in subsurface biomes across the world.

## Data Availability Statement

The original contributions generated for this study are included in the article/Supplementary Material, further inquiries can be directed to the corresponding authors.

## Ethics Statement

Ethical review and approval was not required for the study on human participants in accordance with the local legislation and institutional requirements. Written informed consent for participation was not required for this study in accordance with the national legislation and the institutional requirements.

## Author Contributions

AG and NM conceived the study. AG and NM carried out the bioinformatics analysis. AG, NM, AC, GR, RB, TE-B, MG, and KN interpreted the results and wrote the manuscript. All authors contributed to the article and approved the submitted version.

## Conflict of Interest

The authors declare that the research was conducted in the absence of any commercial or financial relationships that could be construed as a potential conflict of interest.

## Publisher’s Note

All claims expressed in this article are solely those of the authors and do not necessarily represent those of their affiliated organizations, or those of the publisher, the editors and the reviewers. Any product that may be evaluated in this article, or claim that may be made by its manufacturer, is not guaranteed or endorsed by the publisher.

## Supplementary Material

Supplementary Figure 1 Cryptic iron cycling on the surface of an olivine mineral incubated within the Juan de Fuca aquifer (borehole 1301A). The green trapezoid represents the olivine mineral with the chemical formula (Mg^2+^, Fe^2+^)_2_SiO_4_. Oxidation of reduced iron within the olivine mineral by iron-oxidizing bacteria (FeOB) results in the formation of iron(III) oxyhydroxides, and chemical formula Fe(OH)_3_, a by-product of microbial iron oxidation. The iron oxyhydroxides can then be used as terminal electron acceptors by iron-reducing bacteria (FeRB), releasing reduced iron from the mineral. Cyc2 and GACE_1847 are shown as putative iron oxidases and iron reductases, respectively, in this schematic.
