# Supplementary figures and images for: Metagenomic Insights Into the Microbial Iron Cycle of Subseafloor Habitats
Source: Front Microbiol. 2021 Sep 3;12:667944. doi: 10.3389/fmicb.2021.667944 (PMC8446621; doi:10.3389/fmicb.2021.667944)

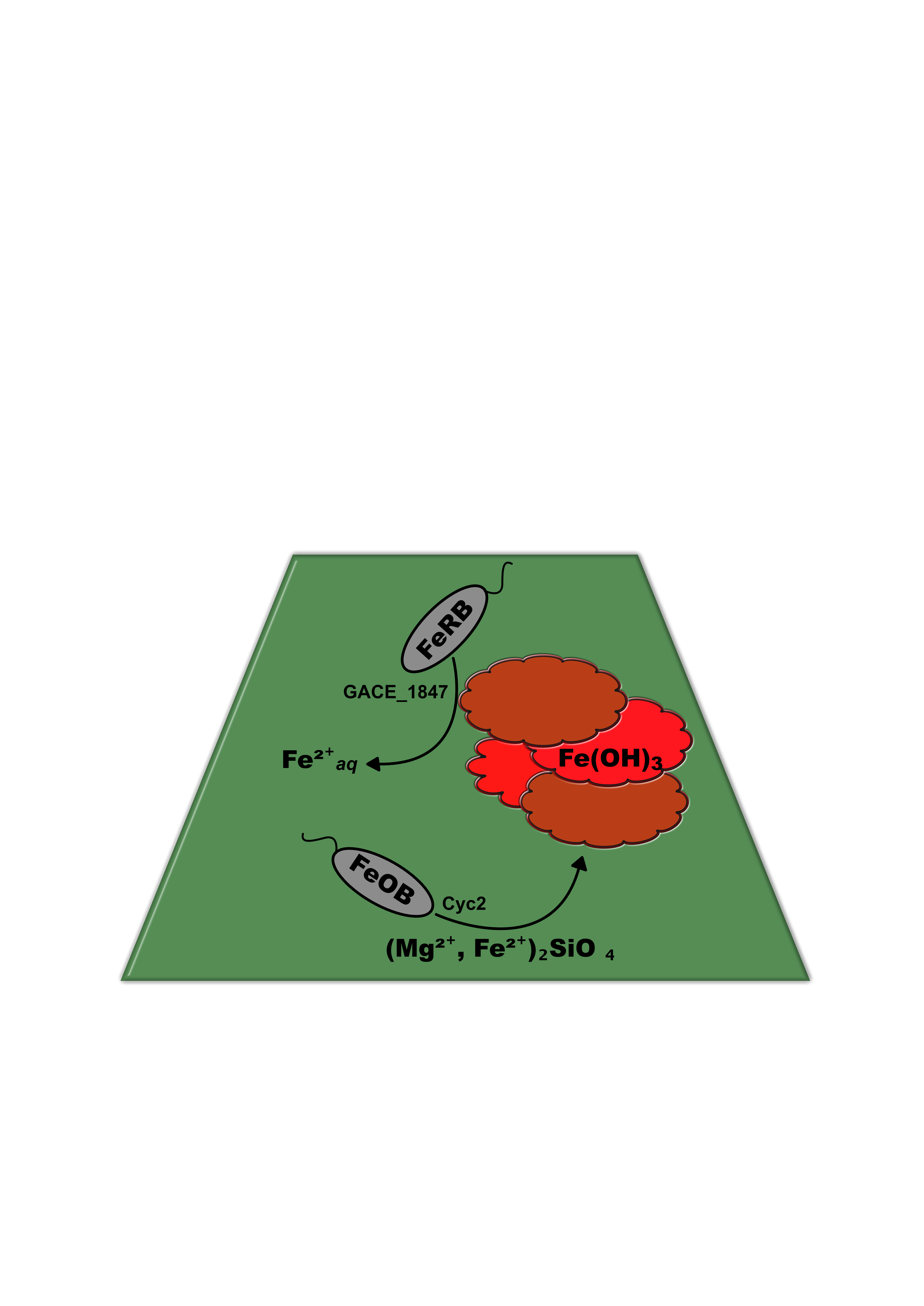

Supplement: Supplementary Figure 1 — Cryptic iron cycling on the surface of an olivine mineral incubated within the Juan de Fuca aquifer (borehole 1301A). The green trapezoid represents the olivine mineral with the chemical formula (Mg2+, Fe2+)2SiO4. Oxidation of reduced iron within the olivine mineral by iron-oxidizing bacteria (FeOB) results in the formation of iron(III) oxyhydroxides, and chemical formula Fe(OH)3, a by-product of microbial iron oxidation. The iron oxyhydroxides can then be used as terminal electron acceptors by iron-reducing bacteria (FeRB), releasing reduced iron from the mineral. Cyc2 and GACE_1847 are shown as putative iron oxidases and iron reductases, respectively, in this schematic. [file Image_1.TIFF]
