# Supplementary material for: Metagenomic Insights Into the Microbial Iron Cycle of Subseafloor Habitats
Source: Front Microbiol. 2021 Sep 3;12:667944. doi: 10.3389/fmicb.2021.667944 (PMC8446621; doi:10.3389/fmicb.2021.667944)
Supplement: Supplementary file 8 [file Data_Sheet_2.DOCX]

**Supplementary Methods**

**Metagenomic Insights into the Microbial Iron Cycle of Subseafloor Habitats**

Arkadiy I. Garber^1^, Ashley B. Cohen^2^, Kenneth H. Nealson^3^, Gustavo A. Ramírez^4,5^, Roman A. Barco^3^, Nancy Merino^6,7^

^1^Arizona State University, School of Life Science, Tempe, AZ, USA

^2^Stony Brook University, School of Marine and Atmospheric Sciences, Stony Brook, NY, USA

^3^University of Southern California, Department of Earth Sciences, Los Angeles, CA, USA

^4^Haifa University, Department of Marine Biology, Haifa, Israel.

^5^Western University of Health Sciences, College of Veterinary Medicine, Pomona, CA, USA

^6^Earth-Life Science Institute, Tokyo Institute of Technology, Tokyo, Japan

^7^Biosciences and Biotechnology Division, Lawrence Livermore National Lab, Livermore, CA, USA

**Correspondence**: agarber4@asu.edu; [merino4@llnl.gov](mailto:merino4@llnl.gov)

*Noteworthy updates to FeGenie’s HMM library and pipeline*

FeGenie was originally designed to identify iron genes and iron gene neighborhoods in relatively complete genomes and high-quality metagenome assemblies (e.g., >5 kbp contigs) (Garber et al., 2020). FeGenie now enables users to infer putative iron genes from fragmented assemblies and (meta)transcriptomes by using the new option --all_results, which bypasses FeGenie’s algorithm and reports all significant HMM hits. The algorithm is designed to reduce the number of false positive hits to genes that are part of broader gene families, which may include non-iron-related functions, by taking into account three factors: the calibrated and optimized bit score cutoffs for each individual HMM, the known operon structures, and the relative gene proximities in iron-related gene neighborhoods. Since the --all_results option will report all matches to FeGenie’s HMM library, this will inevitably produce hits to genes that are likely not associated with iron cycling; thus, when using the --all_results option, we recommend users manually inspect the output, or use the --ref flag to cross-reference each HMM hit to a trusted database of annotated proteins.

FeGenie’s output currently includes the number of heme *c*-binding motifs (e.g. CXXCH, with X representing any amino acid) in each identified gene that is related to iron acquisition, iron redox cycling, iron storage, and magnetosome formation. With the updates, FeGenie will now also report heme *b*-binding motifs (GX[HR]XC[PLAV]G, Li et al., 2011), as well as hematite-binding motifs ([STC][AVILMFYWH][ST]P[ST]; Lower et al., 2007, 2008). Additionally, we added two flags (--heme and --hematite), allowing users to identify and report any and all genes with heme- and hematite-binding motifs. These results are provided in a separate output file.

We also updated FeGenie’s HMM library. Additional genes for iron uptake were added, following the HMM development protocol discussed in Garber et al. (2020). These include two families of metal cation transporters involved with the uptake of Fe(II): the natural resistance-associated macrophage protein (NRAMP; Nevo and Nelson, 2006; Lin et al., 2011) and Zrt- and Irt-related protein (ZIPl4; (Pinilla-Tenas et al., 2011; Woodruff et al., 2018)) families. We note that even though these transporters have been shown to import iron, they are primarily known to transport other substrates. Thus, their interpretation as facilitators of iron acquisition should be taken with caution.

We also added two novel pathways for iron reduction. One of these pathways includes electroactive type IV pili that can be used to carryout extracellular electron transfer to iron (Vargas et al., 2013; Bray et al., 2020). These are thought to facilitate extracellular electron transport from the cytoplasmic membrane to iron minerals via aromatic amino acid residues present on the protein (Vargas et al., 2013). To build this HMM, we used confirmed electroactive (i.e. conductive) pili (from Table S1 in Bray et al., 2020). Identification of electroactive pili is also dependent on sequence features outlined by Bray et al., 2020: at least 9.8% of the sequence must be comprised of aromatic amino acids, no aromatic-free gaps greater than 22 residues, and presence of aromatic amino acids at positions 1, 24, 27, 32, 51, and 57.

The second iron reduction pathway that was added is the five gene operon (GACE_1843-1847), containing multi-heme cytochromes, encoded by *Geoglobus acetivorans* (Mardanov et al., 2015). Genes in this operon encode outer-membrane anchoring domains and hematite-binding sites, (similar to those encoded on the well-characterized extracellular cytochrome ([MtrC)] of *Shewanella oneidensis* MR-1), supporting their role in extracellular electron transfer to insoluble iron-bearing minerals (Mardanov et al., 2015).

A full list of FeGenie updates is listed in **Table S1**.

**References**

Bray, M. S., Wu, J., Padilla, C. C., Stewart, F. J., Fowle, D. A., Henny, C., et al. (2020). Phylogenetic and structural diversity of aromatically dense pili from environmental metagenomes. *Environ. Microbiol. Rep.* 12, 49–57. doi:https://doi.org/10.1111/1758-2229.12809.

Garber, A. I., Nealson, K. H., Okamoto, A., McAllister, S. M., Chan, C. S., Barco, R. A., et al. (2020). FeGenie: A Comprehensive Tool for the Identification of Iron Genes and Iron Gene Neighborhoods in Genome and Metagenome Assemblies. *Front. Microbiol.* 11, 37. doi:10.3389/fmicb.2020.00037.

Li, T., Bonkovsky, H. L., and Guo, J. (2011). Structural analysis of heme proteins: implications for design and prediction. *BMC Struct. Biol.* 11, 13. doi:10.1186/1472-6807-11-13.

Lin, Z., Fernández-Robledo, J.-A., Cellier, M. F. M., and Vasta, G. R. (2011). The Natural Resistance-Associated Macrophage Protein from the Protozoan Parasite *Perkinsus marinus* Mediates Iron Uptake. *Biochemistry* 50, 6340–6355. doi:10.1021/bi200343h.

Lower, B. H., Lins, R. D., Oestreicher, Z., Straatsma, T. P., Hochella, M. F., Shi, L., et al. (2008). In Vitro Evolution of a Peptide with a Hematite Binding Motif That May Constitute a Natural Metal-Oxide Binding Archetype. *Environ. Sci. Technol.* 42, 3821–3827. doi:10.1021/es702688c.

Lower, B. H., Shi, L., Yongsunthon, R., Droubay, T. C., McCready, D. E., and Lower, S. K. (2007). Specific Bonds between an Iron Oxide Surface and Outer Membrane Cytochromes MtrC and OmcA from Shewanella oneidensis MR-1. *J. Bacteriol.* 189, 4944–4952. doi:10.1128/JB.01518-06.

Mardanov, A. V., Slododkina, G. B., Slobodkin, A. I., Beletsky, A. V., Gavrilov, S. N., Kublanov, I. V., et al. (2015). The Geoglobus acetivorans Genome: Fe(III) Reduction, Acetate Utilization, Autotrophic Growth, and Degradation of Aromatic Compounds in a Hyperthermophilic Archaeon. *Appl. Environ. Microbiol.* 81, 1003–1012. doi:10.1128/AEM.02705-14.

Nevo, Y., and Nelson, N. (2006). The NRAMP family of metal-ion transporters. *Biochim. Biophys. Acta BBA - Mol. Cell Res.* 1763, 609–620. doi:10.1016/j.bbamcr.2006.05.007.

Pinilla-Tenas, J. J., Sparkman, B. K., Shawki, A., Illing, A. C., Mitchell, C. J., Zhao, N., et al. (2011). Zip14 is a complex broad-scope metal-ion transporter whose functional properties support roles in the cellular uptake of zinc and nontransferrin-bound iron. *Am. J. Physiol.-Cell Physiol.* 301, C862–C871. doi:10.1152/ajpcell.00479.2010.

Vargas, M., Malvankar, N. S., Tremblay, P.-L., Leang, C., Smith, J. A., Patel, P., et al. (2013). Aromatic Amino Acids Required for Pili Conductivity and Long-Range Extracellular Electron Transport in Geobacter sulfurreducens. *mBio* 4, e00105-13. doi:10.1128/mBio.00105-13.

Woodruff, G., Bouwkamp, C. G., de Vrij, F. M., Lovenberg, T., Bonaventure, P., Kushner, S. A., et al. (2018). The Zinc Transporter SLC39A7 (ZIP7) Is Essential for Regulation of Cytosolic Zinc Levels. *Mol. Pharmacol.* 94, 1092–1100. doi:10.1124/mol.118.112557.
